# Prevalence and Risk Factors of MASLD in Prediabetes and Type 2 Diabetes Mellitus in Belgium and The Netherlands

**DOI:** 10.3390/biomedicines13112821

**Published:** 2025-11-19

**Authors:** Leen J. M. Heyens, Francesco Innocenti, Christophe Van Steenkiste, Mathieu Struyve, Sven M. Francque, Ger H. Koek, Geert Robaeys, on behalf of the MASLD Research Group

**Affiliations:** 1 Limburg Clinical Research Center (LCRC), Faculty of Health and Life Sciences, Hasselt University, 3590 Diepenbeek, Belgium; 2 School of Nutrition and Translational Research in Metabolism (NUTRIM), Maastricht University, 6211 LK Maastricht, The Netherlands; 3 Department of Future Health, Ziekenhuis Oost-Limburg, 3600 Genk, Belgium; 4 Department of Gastroenterology, Ziekenhuis Oost-Limburg, 3600 Genk, Belgium; 5 Department of Methodology and Statistics, Care and Public Health Research Institute (CAPHRI), Maastricht University, 6211 LK Maastricht, The Netherlands; 6 Department of Gastroenterology and Hepatology, AZ Maria Middelares, 9000 Gent, Belgium; 7 Department of Gastroenterology and Hepatology, Antwerp University Hospital, 2000 Antwerp, Belgium; 8 Laboratory of Experimental Medicine and Paediatrics, InflaMed Centre of Excellence, University of Antwerp, 2000 Antwerp, Belgium; 9 Department of Gastroenterology and Hepatology, Maastricht University Medical Center (MUMC+), 6229 HX Maastricht, The Netherlands

**Keywords:** MASLD, prediabetes, type 2 diabetes, Europe, prevalence, risk factors, metabolic dysfunction-associated steatotic liver disease, fibrosis

## Abstract

**Background**: Metabolic dysfunction-associated steatotic liver disease (MASLD) is closely intertwined with glucose metabolism status (GMS). Bi-national comparative epidemiological data are lacking; therefore, this study aimed to provide new insights into MASLD and fibrosis prevalence and risk factors in Belgium, while comparing data from The Netherlands to uncover cross-country differences. **Materials and Methods**: A prospective cohort study (2019–2024) in Belgian primary and secondary care was compared with the Dutch Maastricht Study. Liver fat was measured using CAP (FibroScan^®^), and anthropometric, clinical, and biochemical data were collected. Associations with CAP were analysed using multivariable linear regression, including sex, age, BMI, MetS, high SBP, CVD history, and country. **Results**: A total of 2436 individuals (Belgium and The Netherlands) were screened, of which 1928 were eligible: MASLD with normal GMS (38.3%), prediabetes (19.2%), and type 2 diabetes mellitus (T2DM; 42.5%). Belgian participants with T2DM had higher BMI and prevalence of MASLD. CAP values were significantly associated with female sex (−7.5 dB/m, 95%CI (−11.834; −3.056), *p* < 0.001), BMI (5.184 dB/m, 95%CI (4.627; 5.741), *p* < 0.001), and MetS (13.7 dB/m, 95%CI (8.456; 18.994), *p* < 0.001). Country-specific interactions showed differing effects of high SBP and CVD on CAP between Belgium and The Netherlands, with only the inverse association of CVD history (−10.756, 95%CI (−17.485; −4.027), *p* = 0.002) with CAP in The Netherlands being significant. **Conclusions**: MASLD and fibrosis are common in people with prediabetes and T2DM, underscoring the need for early detection and management. Obesity and metabolic risk were key factors, while a history of CVD appeared protective in the Dutch cohort but not in the Belgian one.

## 1. Introduction

Metabolic dysfunction-associated steatotic liver disease (MASLD) is defined by macrovesicular lipid accumulation in more than five percent of hepatocytes in combination with at least one cardiometabolic risk factor, such as dyslipidaemia, hypertension, and diabetes. It can coexist with other causes of steatosis or chronic liver disease, but when studied in isolation, other classical causes of steatogenesis need to be absent [1]. MASLD in itself encompasses a wide spectrum of clinical conditions, ranging from isolated steatosis to inflammation (metabolic dysfunction-associated steatohepatitis: MASH), liver fibrosis, cirrhosis, and hepatocellular carcinoma (HCC) [2]. It is a heterogeneous disease, and, to date, three distinct MASLD subtypes have been identified based on genetic variants, clinical, and laboratory data [3,4]. The different subtypes have a stronger hepatic genetic component or a stronger metabolic component, be it hepatic or related to adipose tissue dysfunction. Each MASLD has a different effect on, for example, type 2 diabetes mellitus (T2DM), cardiovascular diseases (CVD), dyslipidaemia or fibrosis risk [4]. MASLD is the most common chronic liver disease in the Western world. The global prevalence is currently estimated at 32.4%, which parallels the global increase in obesity and T2DM [5,6,7]. Individuals with MASLD have a 2.2-fold higher risk of developing T2DM, whereas those with T2DM face a 2.0- to 6.0-fold increased risk of developing MASLD, regardless of whether fibrosis is present [8]. This elevated risk is not limited to individuals with fully established T2DM; those with prediabetes are also at an increased risk of developing MASLD [9]. Coexistence of MASLD and prediabetes has an additive effect on the risk of developing T2DM [10].

The pathophysiology of MASLD and diabetes are linked through insulin resistance (IR). Due to our sedentary lifestyles and high caloric intake, adipose tissue expands, triggering adipose tissue IR and inflammation [11,12]. Together with the increased production of reactive oxygen species caused by mitochondrial uncoupling, the dysfunctional adipose tissue, and endotoxins from the gut, a pro-inflammatory climate in the liver is promoted, leading to MASH [13,14,15]. If the pro-inflammatory climate persists, it will ultimately lead to tissue injury and deposition of liver fibrosis [16]. In addition to liver damage, the adipose tissue IR plays a central role in the development of systemic IR. The systemic IR results in the inability of peripheral tissues, such as muscle, to effectively respond to insulin, thereby hindering glucose uptake and regulation [17]. Consequently, this cascade contributes significantly to the onset of prediabetes and T2DM by exacerbating hyperglycaemia and metabolic dysregulation.

Although the association between MASLD, prediabetes, and T2DM is well established, data on MASLD and fibrosis prevalence in individuals with T2DM in Belgium are still scarce, and no data exist for those with prediabetes. Therefore, the aim of this study was to contribute new insights into MASLD and fibrosis prevalence in Belgium, while also enhancing understanding of risk factors within this population. Furthermore, by comparing findings with data from The Netherlands, this study explores potential differences in prevalence and associated risk factors between two neighbouring European countries.

## 2. Materials and Methods

### 2.1. Study Design

Prospective data from the following cross-sectional cohort studies were used: the SCREEFLAN study (NCT04647409, Belgium), the PAD2ZOL (NCT06445335, Belgium), NADIA (NCT04999124, Belgium), and The Maastricht Study (The Netherlands) [18]. These studies were conducted according to the Helsinki Declaration after approval by the Ethics Committee of Hasselt University (CME2020 019), of Antwerp University Hospital (19/44/495), Maria Middelares (MMS.2019.019), the Committee Medical Ethics of Ziekenhuis Oost-Limburg (CTU2020015) and by the institutional medical ethical committee (NL31329.068.10) and the Ministry of Health, Welfare, and Sports of The Netherlands (Permit 131088-105234-PG). Good clinical practice (GCP) guidelines were followed throughout the study. Written informed consent was obtained from all participants.

### 2.2. Participants

Participants were divided into three groups: (1) participants who had a normal glucose metabolism status (GMS) in combination with MASLD (defined as a CAP value above 248 dB/m combined with one cardiometabolic criterion [19], (2) people with prediabetes with or without MASLD and (3) participants who were diagnosed with T2DM with or without MASLD. Belgian participants were either recruited on an ongoing basis by their primary care providers or by their endocrinologists in secondary care (Supplementary Table S1). Participants from Belgium were eligible if they were older than 18 years, able to understand Dutch, and had no excessive alcohol use (more than 2 or 3 glasses of alcohol per day for women or men, respectively). Individuals with a known history of other liver diseases, i.e., hepatitis B, hepatitis C, autoimmune hepatitis, primary biliary cholangitis, haemochromatosis, Wilson’s disease, or Alpha 1 antitrypsin deficiency, were excluded. Other exclusion criteria were secondary causes of steatosis or drug-induced liver injury, which included the use of medications such as amiodarone, tamoxifen, and methotrexate.

Data from The Maastricht Study, a prospectively designed, population-based observational cohort study in The Netherlands, were used. In brief, the study focused on the aetiology, pathophysiology, complications and comorbidities of T2DM. Participants were recruited through mass media campaigns and from the municipal registries and the regional Diabetes Patient Registry via mailings [18]. For the current analysis, the Dutch participants were also excluded if excessive alcohol use, a known history of other liver diseases, secondary causes of steatosis, or drug-induced liver injury were present.

### 2.3. Glucose Metabolism Status

For Belgian participants, a diagnosis of T2DM was based on the available diagnosis in the electronic patient records (EPR) or the use of glucose-lowering medication. Prediabetes was based on impaired fasting glucose (110–125 mg/dL) or HbA1c between 6 and 6.4% [20] (Supplemental Table S2). Belgian participants without any of the above-mentioned deviations in glucose metabolism were classified as having normal glucose metabolism.

For The Netherlands, after an overnight fast, participants, except those who used insulin or had a fasting plasma glucose (FPG) concentration above 11.0 mmol/L, underwent an oral glucose tolerance test (OGTT) post ingestion of a 75 g glucose drink (Supplemental Table S2). Based on FPG, 2-h-FPG, and glucose-lowering medication use, GMS was determined as normal glucose metabolism (NGM), prediabetes (impaired fasting glucose, impaired glucose tolerance, or both), or T2DM in accordance with the World Health Organisation 2006 criteria [21] (Supplemental Table S2).

### 2.4. Data Collection

Anthropometric data (waist circumference, weight, height, and blood pressure (BP)), smoking status (never, former, current), alcohol use, medical background, medication use, and demographic data were collected on the day of evaluation for the Belgian cohort. Venous blood samples were taken within one month of the FibroScan^®^ (Echosens, Paris, France) measurement. Missing anthropometric data, laboratory values (in this case, within 6 months of the FibroScan^®^ measurement), medical history, and medication use were collected retrospectively from EPR. For Dutch participants, medical history was self-reported and not verified against the EPR. Medication use was documented during a structured interview with the researcher, and laboratory parameters were obtained from venous blood samples. A body mass index (BMI) of <25 kg/m^2^, 25–30 kg/m^2^, and >30 kg/m^2^ was considered as normal weight, overweight, and obesity, respectively [22,23]. Furthermore, metabolic syndrome (MetS) diagnosis was based on the International Diabetes Federation consensus [24]. High systolic (SBP) and diastolic blood pressure (DBP) were based on the cardiometabolic criteria outlined in the MASLD definition [1]. People were regarded as having a history of CVD, if they reported that they have ever had at least one of the following conditions/treatments: (i) myocardial infarction, (ii) cerebrovascular infarction and/or haemorrhage, (iii) percutaneous artery angioplasty of the coronary arteries, abdominal arteries, peripheral arteries or carotid artery, (iv) vascular surgery on coronary arteries, abdominal arteries, peripheral arteries or carotid artery.

### 2.5. Liver Stiffness and Steatosis Measurements

As a surrogate for liver fibrosis, liver stiffness was measured using Vibration-Controlled Transient Elastography (VCTE^TM^), and liver steatosis was assessed using Controlled Attenuation Parameter (CAP^TM^). VCTE and CAP were measured utilising a FibroScan^®^ 430+ mini in the right liver lobe by intercostal approach. Subjects were asked to be in a fasting state for at least 3 h before the examination and were placed in a supine position with their arms in maximal abduction. For screening, the M probe (3.5 MHz) or the XL probe (2.5 MHz) was selected based on the device’s indication. VCTE and CAP values were considered reliable when the interquartile range (IQR) was equal to or less than 30% of the median Liver Stiffness Measurement (LSM) value (IQR/med), and at least ten measurements were performed. A CAP value of >248 dB/m was considered as having steatosis [19]. Fibrosis stages based on VCTE values are categorised as follows: a VCTE value of <8.0 kPa, ≥8–12 kPa, or >12 kPa was considered as having no to little fibrosis (F0–F1), significant fibrosis (F2), or advanced fibrosis and cirrhosis (F3–F4), respectively [19].

### 2.6. Statistical Analysis

Statistical analyses were performed using Statistical Package for Social Sciences (SPSS) (IBM Corp. Released 2019. IBM SPSS Statistics for Windows, Version 26.0. Armonk, NY, USA: IBM Corp.) and R Version 4.5.1 (R Core Team. Released 2025. R Foundation for Statistical Computing. Vienna, Austria). Categorical variables were presented as percentages, along with their absolute values. The Shapiro–Wilk and Kolmogorov–Smirnov tests were used to test for normality of continuous variables. Numeric variables are presented as mean with standard deviation or median with interquartile range (IQR). For variables with a normal distribution, the t-test was used to compare the means of two groups. For non-normally distributed data, the non-parametric Mann–Whitney U test and the Kruskal–Wallis test were used to compare two or more groups, respectively. The number of subjects included in every analysis is designated by ‘n’. A linear regression model containing the following variables: age, sex, BMI, high SBP, MetS, smoking status, and a history of CVD, was used to determine the risk factors for steatosis (expressed as a continuous CAP value). Two-way and three-way interaction terms were included between groups (MASLD, prediabetes, T2DM), country, and selected risk factors, in addition to the main effects of the covariates. To reduce multicollinearity, age and BMI were centred at their respective means, 65.63 years and 29.2 kg/m^2^, for the total cohort. BMI and sex were included as covariates without interaction terms, as each was considered an independent predictor not expected to interact meaningfully with either group or country [25,26,27,28,29,30]. Due to the limited availability of data on medication use, only a sensitivity analysis was conducted, which included an additional adjustment for the use of statins. Backward elimination selection was performed starting from the three-way interaction model, respecting the hierarchy principle, to determine the final model. Model assumptions were assessed using QQ plots for residual normality, residuals-versus-predicted-values plots for homoscedasticity, Variance Inflation Factors (VIF) > 10 for multicollinearity, Cook’s distance ≥ 1 for influential outliers, and tests of quadratic effects for linearity of quantitative predictors. Estimated marginal means were used to assess differences in CAP by CVD status within the country after adjusting for GMS, age, sex, BMI, and high SBP, MetS, and statin use.

## 3. Results

### 3.1. Steatosis and Fibrosis in the Bi-National Cohort

**Figure 1:**
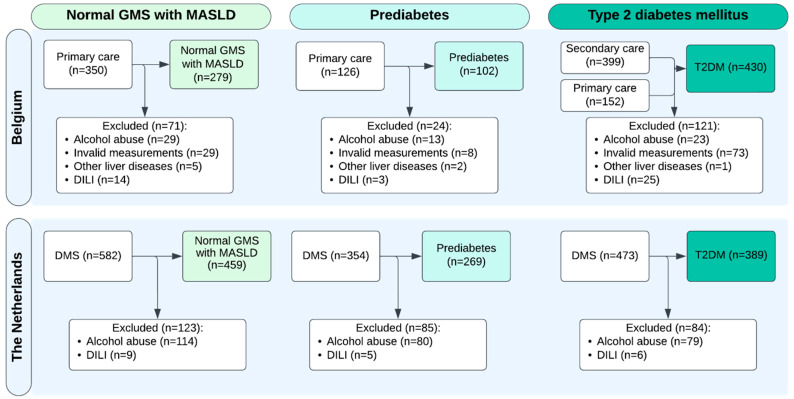
Flowchart of inclusion. Abbreviations: DILI: drug-induced liver injury, DMS: de Maastricht Study, T2DM: type 2 diabetes mellitus.

In total, 2436 people were screened in Belgium (42.2%) and The Netherlands (57.8%). Of those, 1928 (79.2%) were eligible according to the inclusion criteria (Figure 1). For the total cohort, the median age was 67 [IQR 13] years, 1080 (56.0%) were male, and the median BMI was 28.4 [5.9] kg/m^2^ (Table 1). The study population was stratified according to GMS, resulting in three groups: a group with normal GMS and MASLD (n = 738, 38.3%), prediabetes with or without MASLD (n = 371, 19.2%), and a T2DM group with or without MASLD (n = 819, 42.5%) (Figure 1).

MASLD was more prevalent in Belgian individuals with T2DM (n = 354, 82.3%) compared to those in The Netherlands (n = 290, 74.6%, *p* = 0.007) (Table 1). In prediabetes, a trend was observed for MASLD prevalence (n = 74, 72.5% vs. n = 173, 64.3%, *p* = 0.133), with Belgium having the highest prevalence. Accordingly, CAP values were higher in the Belgian T2DM group (312 [81.0] vs. 294 [93.0] dB/m, *p* < 0.001) but not the prediabetes group (*p* = 0.674). Liver stiffness, measured via VCTE, was highest in Belgian T2DM individuals (6.6 [5.1] kPa), with the highest prevalence of F2 (n = 166, 38.6%) and F3–F4 (n = 78, 18.1%) (Table 1). Whereas the Dutch prediabetes (n = 4, 1.5%) participants had a fibrosis prevalence similar to the normal GMS with MASLD group (n = 6, 1.4%).

### 3.2. Characteristics and Differences in the Bi-National Cohort

The results showed that participants with T2DM and prediabetes were consistently older within the Dutch cohort compared to the Belgian cohort, particularly in the T2DM group (71 [10.0] years vs. 62 [12.0] years in the Belgian cohort, *p* < 0.001) (Table 1). Male sex was more common in T2DM groups, with the highest proportion (*p* = 0.001) in the Dutch T2DM cohort (n = 268, 68.9% vs. n = 147, 57.9%). BMI was significantly higher in Belgian participants across all groups, with Belgian individuals with T2DM showing the highest median BMI of 31.0 [7.4] kg/m^2^. Waist circumference followed similar trends, except between men with T2DM, where no significant difference was found when comparing countries.

No significant differences were observed between countries or groups concerning having a history of CVD. High SBP was significantly more prevalent in Belgium, except in the T2DM group. The prevalence of MetS was markedly higher in T2DM individuals, especially in The Netherlands (n = 338, 86.9% vs. n = 347, 80.7%, *p* = 0.001), and remained elevated but not significantly different in prediabetes groups. Cardiometabolic parameters, including HDL, LDL, and total cholesterol levels, were lower in the Belgian MASLD group and T2DM participants (Table 1).

Medication use patterns aligned with GMS, with all types being used most frequently by people with T2DM, regardless of country. Glucose-lowering medications were used by 362 (94.8%) T2DM participants in Belgium, while in The Netherlands, the use was significantly lower (*p* < 0.001). More in detail, people with T2DM in Belgium used glucagon-like peptide (GLP)-1 receptor agonists, insulin, and metformin more frequently compared to the Dutch T2DM cohort (Supplemental Table S3). Anti-hypertensive and lipid-lowering drug use followed similar trends, with the highest use in the T2DM group and the lowest in the normal GMS MASLD group.

### 3.3. Risk Factor Analysis

To determine the MASLD-associated risk factors, the final model included sex, BMI (centred), BMI^2^, presence of MetS, age (centred), high SBP, and a history of CVD, with the latter three having interaction terms with GMS or country. The effect of each predictor on CAP should therefore be interpreted as adjusted for the other variables. Assumption checks indicated that residuals were approximately normal and homoscedastic, with no collinearity or influential outliers detected. Non-linearity was observed only for BMI. The estimated predicted CAP value of the final model was 289.072 dB/m, for a reference patient defined as a 65.63-year-old male with normal GMS and MASLD, a BMI of 29.20 kg/m^2^, no MetS, no high SBP, no history of CVD, and residing in Belgium (Table 2 and Tables S2–S5).

Sex was significantly associated with CAP, with female patients showing 7.445 dB/m lower values than males while accounting for the other covariates (Table 2). The adjusted effect of BMI on CAP was non-linear and therefore modelled with a quadratic term (Supplemental Figure S2). Specifically, CAP increased with BMI, but the rate of increase diminished at higher BMI values. The increase was steepest between BMI values of approximately 16 kg/m^2^ and 35 kg/m^2^ and slowed down thereafter. The presence of MetS was significantly associated with a higher CAP, with patients exhibiting MetS having CAP values 13.725 dB/m greater than those without MetS, when adjusting for other factors (Table 2).

The analysis showed that the adjusted effect of age on CAP varied across GMS groups (Supplemental Tables S4–S7). For individuals with normal GMS and MASLD, this difference was not statistically significant (Table 2), as only those with a CAP value above 248 dB/m were selected. This selection criterion may have excluded part of the spectrum, which could potentially influence statistical significance. While there was a small, statistically significant association between age and CAP for individuals with prediabetes (−0.663 dB/m per year, *p* = 0.019) and those with T2DM (−0.755 dB/m per year, *p* = 0.003) (Table 2 and Tables S4–S7), Consequently, the adjusted difference in CAP between prediabetes and normal GMS with MALSD patients decreased with age. CAP was lower in normal patients at younger ages, but this trend reversed as people got older, with prediabetes patients exhibiting lower CAP thereafter (Supplemental Figure S1). A similar pattern was observed for individuals with T2DM compared to those with normal GMS MASLD. This relationship showed that normal patients had lower CAP values up to about 50 years of age, after which people with T2DM had lower CAP values. When comparing people with T2DM to prediabetes, the adjusted difference indicated a mild decrease in the difference with age, but a consistently higher CAP in T2DM patients. As for the effect of prediabetes and T2DM compared to those with normal GMS and MASLD, the analysis showed that patients with prediabetes had a mean CAP that was 25.342 dB/m lower at the mean age, while those with T2DM had a CAP that was 10.422 dB/m lower (Supplemental Tables S4–S7). Sensitivity analysis in which statin use was added to the regression model showed no difference in results (Supplemental Tables S8–S11).

Country-specific effects further modified the adjusted associations between high SBP, history of CVD, and CAP. Although not significant (*p* = 0.106), the results of the analysis indicated that having high SBP was associated with a 6.455 dB/m increase in CAP among Belgian patients (Table 2). On the other hand, though also not significant (*p* = 0.080), in The Netherlands, having a high SBP was associated with a 5.249 dB/m lower CAP. Likewise, Dutch patients with a history of CVD had significantly lower CAP values than those without (−10.756 dB/m, *p* = 0.002). In contrast, among Belgian patients, CVD history was not significantly associated with CAP (*p* = 0.295), although CAP values were 4.518 dB/m higher in those with the condition than in those without (Table 2). Marginal means showed that the CAP means were not different based on the history of CVD within the country (Supplemental Tables S12 and S13).

## 4. Discussion

Depending on the country, the prevalence of MASLD in prediabetes and T2DM ranged from 64.3% to 72.5% and 74.6% to 82.3%, respectively, in The Netherlands and Belgium (Supplementary Figure S3). We hence report a high prevalence of MASLD in prediabetes and even more in patients with T2DM. A recent meta-analysis reported a pooled MASLD prevalence of 69% in T2DM for Western Europe, which is consistent with our findings for The Netherlands. In contrast, we observed a statistically higher prevalence in Belgium [7]. The higher prevalence observed in Belgium may be partly explained by selection bias within the cohort, as some individuals with T2DM were recruited from the secondary care gastroenterology department, where they presented with gastrointestinal complaints. However, in line with our findings, Kim et al. found a MASLD prevalence of 84.5% among individuals with T2MD using data from the NHANES cohort [31]. Another contributing factor could be the diagnostic method applied in the meta-analysis, as variations in methodology can lead to differing prevalence estimates [7]. For example, one of the studies from the meta-analysis found that MASLD was present in 59.6% of the Italian T2DM population. This study utilised the Fatty Liver Index (FLI), a score based on routinely available parameters, to calculate the prevalence. However, the FLI is less accurate in diagnosing steatosis than the CAP [32,33]. The T2DM patients in the Edinburgh study in Scotland, on the other hand, were screened for steatosis using an ultrasound (US), and a steatosis prevalence of 42.6% was found [34]. Ultrasound B-mode imaging enables the radiologist to subjectively estimate the amount of liver steatosis by comparing the liver brightness to that of the kidney [35]. However, the reported sensitivity for mild cases of steatosis is low, and the visualisation can be impaired due to abdominal gas or obesity [35,36]. Regarding prediabetes, a large Korean cohort (n = 985) found a MASLD prevalence of 33% based on US [37]. Although lower than the rates in Belgium or The Netherlands, MASLD prevalence in prediabetes remained significantly higher than in the normal GMS group of the Korean study [37]. Corbin et al. observed an 84.1% prevalence of MASLD using the Hepatic Steatosis Index and 69.2% according to the NAFLD Liver Fat Score in patients with prediabetes [38]. Similarly to our results, Kim et al. found the prevalence of MASLD to be 61.1% when using CAP (≥263 dB/m) [31]. Although prevalence estimates vary depending on the diagnostic method used or the characteristics of the cohort (i.e., level of diabetes control, type of anti-diabetic drugs used), MASLD remains markedly common in individuals with T2DM and prediabetes compared to the general population [5,6].

We also report a non-negligible prevalence of fibrosis, including advanced fibrosis, particularly in patients with T2DM. Specifically, 20.5% of T2DM participants in Belgium had F2, and 18.1% had F3–4. In The Netherlands, the corresponding figures were 6.2% with F2 and 3.6% with F3–4. A study performed in Romania with 392 T2DM patients, of whom 82.7% had steatosis on US, found a prevalence of 18.8% of ≥F2 in patients with steatosis, and 13.8% patients had cirrhosis diagnosed with VCTE^TM^ [39]. Two other studies found F2 prevalences of 16.1% (VCTE^TM^ determined) and 16.7% (magnetic resonance elastography (MRE) determined), respectively [37,40]. These percentages are similar to those from Belgium, but not The Netherlands, where the prevalence was lower. We hypothesise that this difference is attributable to the design of the Dutch cohort. Dutch people with more advanced T2DM did not present themselves for The Maastricht Study, and there is also a small percentage (5.3%) of people with newly diagnosed T2DM. This is reflected in the number of people who use insulin, which is significantly lower in The Netherlands compared to Belgium. Fibrosis was also detected in individuals with prediabetes, albeit at lower levels than in those with T2DM, and comparable to the individuals with normal GMS and MASLD in Belgium and The Netherlands. Within a prediabetes Korean cohort, 3.7% had fibrosis stage F2 and 0.7% had F3–4, consistent with our findings in the Dutch cohort [37]. The prevalence of F3–4 was 3.3% according to the FIB-4 score and 1.1% according to the APRI, as observed by Corbin et al. in people with prediabetes, which was significantly lower than that of those with T2DM but higher than that of those with normal GMS [38]. With respect to liver fibrosis, our findings indicate that it is more prevalent in individuals with T2DM than in those with prediabetes or MASLD alone. The results generated by our study reinforce the recommendations for fibrosis screening in individuals with T2DM, as outlined in the most recent American Diabetes Association (ADA) consensus [41]. For prediabetes, it is recommended by the ADA to screen for fibrosis after a risk stratification, particularly in the presence of obesity [41]. According to the guidelines of the European Study of the Liver and the American Association for the Study of the Liver, screening for fibrosis in T2DM is also recommended; however, prediabetes is scarcely addressed, and the guidelines are unclear [19,42].

With regard to the country-specific interactions in the regression analysis, though only a history of CVD is significant, the findings clearly represent a trend when comparing the effect of CVD history and high SBP between Belgium and The Netherlands (Supplementary Figure S3). Whereas having high SBP and a history of CVD is protective in The Netherlands, it was the opposite in Belgium. CVD history and high SBP are established risk factors for MASLD [43,44]. However, considering the heterogeneity of MASLD, one of the subtypes described by Stefan et al. is characterised by a strong hepatic genetic component and correlates with a low or absent risk of CVD but increased risk of T2DM [4]. This subtype may be more prevalent in the Dutch cohort, while in the Belgian cohort, it could be one of the subtypes with a stronger metabolic component [4]. We also hypothesised that this difference may stem from the stronger emphasis on preventive healthcare in The Netherlands compared to Belgium. Therefore, we believe that Dutch people who have had CVD or are diagnosed with high SBP are better guided towards a healthy lifestyle and experience the benefit with a lower risk for MASLD. To illustrate the focus of the Dutch system on prevention, our study cohort revealed a higher prescription rate for glucose and lipid-lowering medications in Belgium compared to The Netherlands across all subgroups, suggesting a greater emphasis on treatment rather than prevention. Another explanation for the lower prescription rates may relate to the predominant MASLD subtype in the analysed cohort. Individuals with a MASLD subtype driven primarily by hepatic genetic factors tend to exhibit less dyslipidaemia and therefore require fewer lipid-lowering medications [4]. Conversely, if the metabolically driven subtype is more prevalent in Belgium, these individuals are more likely to develop T2DM and consequently require more glucose-lowering therapy. The lower prescription rates may also reflect the strict adherence to general practice guidelines in The Netherlands. These guidelines tend to be very conservative in terms of medication prescription, and adherence to them is relatively high. In Belgium, there are often no guidelines (or they refer to the Dutch ones), there is a more liberal prescription of drugs, and adherence to the guidelines is less stringent. Interestingly, although the prescription rates of anti-hypertensives for prediabetics were similar in both countries, in Belgium, a higher SBP was observed. This difference could be the result of the study’s methodology. During the study visit in Belgium, only one BP measurement was taken, while in The Netherlands, three were taken, of which the average was used [18].

The regression analysis also identified sex, BMI, and MetS as significant risk factors of steatosis. The presence of MetS was associated with increased steatosis, regardless of other risk factors, and female sex was associated with lower steatosis values, as previous research had indicated [45,46,47]. Although BMI is not a perfect measure of body composition as it only reflects the ratio of total body weight to height and cannot distinguish between adipose and lean (including muscle) tissue, and the different adipose tissue depots, we do see a similar non-linear relationship with steatosis as metrics that do reflect obesity better [27,48,49]. A plateau was observed for people with a high BMI. It is thought that at very high BMI levels, most susceptible individuals are likely to have already developed MASLD, while the remaining population may possess protective factors that mitigate the additional effect of further BMI increases [27]. Furthermore, it is hypothesised that if people have lower body fat levels, fat deposition tends to happen preferentially as visceral adipose tissue (VAT) [50]. VAT is metabolically active and known to play a role in the development of MASLD through the release of free fatty acids (FFAs) into the portal circulation [51,52,53]. Even modest increases in VAT, even in individuals with a low BMI, can increase IR and steatosis, thereby increasing the risk for MASLD [53]. When total body fat further accumulates, it mostly happens in the subcutaneous fat depots. As subcutaneous fat is less metabolically active than VAT, the accumulation does not result in excess FFAs and corresponding hepatic lipotoxicity [53,54]. The changes in the different fat depots could also explain the observed non-linear relationship.

Interestingly, the regression analysis also showed that the effect of age on steatosis was minimal for prediabetes and T2DM. In contrast, the interaction between age and GMS revealed significant age-dependent declines in steatosis among those with prediabetes and T2DM. Steatosis values in patients with prediabetes or T2DM were paradoxically lower than those with normal GMS at older ages. These results were similar to a Japanese study, where they found MASLD detected by computed tomography (CT) to be associated with younger age in T2DM individuals [55]. Also, a Chinese study found the prevalence of MASLD to be decreasing with older age among T2DM patients [53]. It has been hypothesised that several mechanisms may underlie this paradoxical finding. First, survivor bias may be at play, as individuals with more severe MASLD and T2DM may experience earlier morbidity or mortality compared to normal GMS, and thus be underrepresented in older age groups [56]. Still, van Kleef et al. showed no association between mortality and MASLD in an elderly population [57]. Moreover, it is worth noting that Targher et al. did not observe an age-related decline in steatosis [58]. The observed finding in our study cohort could result from the additive effect of having T2DM and MASLD rather than solely MASLD [56]. Secondly, we hypothesised that steatosis may decline in individuals with progressive liver disease as fibrosis advances, leading to lower steatosis values in older people despite worsening hepatic disease [59].

A major strength of our study is its comprehensive evaluation of MASLD and liver fibrosis across a normal GMS with MASLD, prediabetes, and T2DM with or without MASLD population in two neighbouring European countries, allowing cross-country comparisons. Moreover, the large sample size and inclusion of both patients with prediabetes and T2DM individuals provide strong evidence for the high prevalence of MASLD and fibrosis in these populations. The study also highlights important age- and country-specific interactions, offering nuanced insights into how demographic and healthcare-related factors influence liver outcomes. Finally, the findings align with and reinforce international recommendations for MASLD and fibrosis screening in individuals with prediabetes and T2DM, emphasising the clinical relevance and translational impact of the results. Despite the strengths of the study, there are limitations. First, the lack of liver biopsy, the gold standard for diagnosing liver fibrosis and other relevant features of MASLD, is a potential limitation [60]. Secondly, selection bias could not be avoided entirely in the Belgian and Dutch cohorts. An important strength of the Belgian study is its primary care focus; however, unlike the population-based Maastricht Study, recruitment followed different procedures. Since Belgian participants were recruited through primary care, meaning they were already under medical surveillance or seeking treatment, the sample likely represents a more advanced or severe population, whereas the Dutch approach captures a broader, less biased cohort. Third, we have limited data on medication use available from some participants of the SCREEFLAN study. Hence, we were only able to correct for statin use in the multivariable regression model. Lastly, no data on liver enzymes were available for the Dutch cohort and could therefore not be compared.

## 5. Conclusions

MASLD is highly prevalent in individuals with prediabetes and especially T2DM in Belgium and The Netherlands. While fibrosis was less common in prediabetes than in T2DM, its presence remains clinically relevant and highlights the need for systematic screening. Predictors of steatosis were sex, BMI, and MetS, while age-related interactions suggest complex disease dynamics. Finally, country-specific differences further indicate that healthcare systems with a stronger preventive focus, such as in The Netherlands, may mitigate the impact of traditional risk factors like CVD history and high SBP.

## Figures and Tables

**Table 1:** Characteristics of the total cohort and subcohorts stratified by country.

Characteristic	Total Cohort (n = 1928)	Belgium (n = 811)			The Netherlands (n = 1117)			*p* ^1^	*p* ^2^	*p* ^3^
		Normal GMS with MASLD (n = 279)	Prediabetes (n = 102)	T2DM (n = 430)	Normal GMS with MASLD (n = 459)	Prediabetes (n = 269)	T2DM (n = 389)			
Demographics										
Age (years)	67 [13.0]	62 [17.0]	64.0 ± 10.5	62 [12.0]	67 [12.0]	70.0 [10.0]	71 [10.0]	α	α	α
Sex (male)	1080 (56.0)	147 (52.7)	49 (48.0)	249 (57.9)	226 (49.2)	141 (52.4)	268 (68.9)	-	-	α
BMI (kg/m^2^)	28.4 [5.9]	29.9 [5.6]	29.8 ± 5.0	31.0 [7.4]	27.0 [4.3]	27.3 [5.0]	28.0 [5.1]	α	α	α
Waist circum. (cm)										
Male	104.0 [14.9]	106.5 [13.1]	107.0 ± 12.6	107.0 [16.9]	99.1 [10.0]	101.7 ± 10.0	106.3 [13.7]	α	α	-
Female	97.0 [19.0]	101.3 ± 13.1	99.2 ± 11.9	106.5 [21.0]	91.4 [14.5]	91.9 ± 11.6	96.8 ± 12.0	α	α	α
Blood pressure *										
High SBP	1238 (64.2)	194 (69.5)	72 (70.5)	291 (73.9)	237 (51.6)	155 (57.6)	289 (74.5)	α	α	-
High DBP	633 (32.8)	138 (49.5)	50 (49.0)	189 (48.0)	105 (22.9)	62 (23.1)	89 (23.0)	α	α	α
Smoker status										
Never	867 (45.0)	145 (52.0)	56 (54.9)	196 (45.7)	214 (46.6)	106 (39.4)	150 (38.6)	α	α	α
Former	899 (46.7)	107 (38.4)	37 (36.3)	171 (39.9)	222 (48.4)	148 (55.0)	214 (55.0)			
Current	161 (8.4)	27 (9.7)	9 (8.8)	62 (14.15)	23 (5.0)	15 (5.6)	25 (6.4)			
**Metabolic health**										
CVD history	577 (29.9)	36 (12.9)	13 (12.7)	112 (26.0)	79 (17.2)	53 (19.7)	284 (27.0)	-	-	-
Metabolic syndrome	1214 (62.9)	161 (57.7)	70 (68.6)	347 (80.7)	137 (29.8)	161 (59.9)	338 (86.9)	α	-	α
**Laboratory values**										
HDL-cholesterol (mg/dL)	50.3 [19.3]	49.0 [18.0]	58.3 ± 12.4	44.0 [15.0]	58 [23.2]	58 [19.3]	46.4 [15.5]	α	-	α
LDL-cholesterol (mg/dL)	102.9 [56.7]	111.0 [45.5]	120.6 ± 30.8	71.0 [44.9]	123.8 [46.4]	125.8 ± 35.3	85.1 [42.5]	α	-	α
Total cholesterol (mg/dL)	181.8 [61.9]	190.0 [51.3]	204.4 ± 31.4	151.0 [49.0]	203.3 ± 40.8	210.0 ± 39.7	154.7 [54.1]	α	-	α
Triglycerides (mg/dL)	124.9 [87.7]	136.0 [93.0]	130 [59.0]	149.5 [118.5]	106.3 [62.9]	121.3 [70.9]	134.6 [89.9]	α	-	-
HbA1c (%)	5.9 [1.5]	5.8 [0.7] ^†^	6.0 ± 0.3	7 [1.2]	5.4 [0.5]	5.7 [0.5]	7.0 [1.3]	α	α	-
Serum creatinine (mg/dL)	0.87 [0.26]	0.84 [0.23]	0.77 ± 0.13	0.89 [0.34]	0.84 [0.23]	0.78 [0.16]	0.90 [0.28]	-	-	-
Thrombocytes (×10^9^/L)	231.0 [77.0]	247.5 [86.8]	262.6 ± 55.7	227 [85.8]	231.5 [71.0]	250.0 [71.0]	224 [77.8]	α	-	-
**Medication** use										
Glucose-lowering medication *	648 (36.5)	7 (3.5) ^††^	2 (2.7)	362 (94.8)	0 (0.0)	0	277 (71.2)	α	α	α
Anti-hypertensives **	966 (54.5)	83 (41.3)	34 (45.9)	288 (75.6)	152 (33.1)	129 (48.0)	280 (72.0)	α	-	-
Lipid-modifying medication ***	926 (50.2)	104 (45.2)	47 (54.7)	321 (78.3)	106 (23.1)	89 (33.1)	259 (66.6)	α	α	α
Liver parameters										
VCTE^TM^ (kPa)	5.3 [2.4]	5.5 [2.3]	4.9 [2.1]	6.6 [5.1]	4.6 [1.7]	4.4 [1.7]	5.3 [2.3]	α	-	α
F2	165 (8.6)	35 (12.5)	12 (11.8)	88 (20.5)	3 (0.7)	3 (1.1)	24 (6.2)	α	α	α
F3–4	99 (5.1)	3 (1.1)	0 (0.0)	78 (18.1)	3 (0.7)	1 (0.4)	14 (3.6)	-	-	α
CAP^TM^ (dB/m)	290.0 [72.0]	303.0 [62.0]	270.5 ± 50.1	312 [81.0]	283.0 [42.0]	265.5 ± 58.3	294 [93.0]	α	-	α
MASLD	1629 (84.5)	100%	74 (72.5)	354 (82.3)	100%	173 (64.3)	290 (74.6)	N.A	-	α

Data are presented as mean ± standard deviation, median [interquartile range], or number (%). *p*^1^: difference between normal GMS with MASLD groups, *p*^2^: prediabetes comparison, *p*^3^: T2DM groups compared. † Bias for HbA1c (%), it was only determined in people with suspicion of prediabetes or T2DM in the MASLD group from Belgium. ^††^ In the group of MASLD in Belgium, the following types of glucose-lowering medication were used: metformin (n = 4/7), GLP-1 receptor agonist (n = 2/7), and SGLT-2 inhibitor (n = 1/7). Missing data on medication use in Belgium over all subcohorts: * n = 152, ** n = 155, and *** n = 85. Abbreviations: BMI: body mass index, HbA1c: haemoglobin A1c, Waist circum.: waist circumference, HDL: high-density lipoprotein, LDL: low-density lipoprotein, CVD: cardiovascular disease, SBP: systolic blood pressure, DBP: diastolic blood pressure, T2DM: type 2 diabetes mellitus, CAP: controlled attenuation parameter, VCTE: vibration-controlled transient elastography.

**Table 2:** Linear regression analysis for risk factors of steatosis in the prediabetes, T2DM, and normal GMS MASLD population (n = 1860).

Variable	β	SE	*p*	95% CI
**Variables not part of any interactions**				
Intercept	289.072	4.317	α	280.605; 297,548
Sex	−7.445	2.238	α	−11.834; −3.056
BMI	5.184	0.284	α	4.627; 5.741
MetS	13.725	2.686	α	8.456; 18.994
**Effect of interaction terms on CAP**				
BMI^2^	−0.077	0.031	α	−0.138; −0.016
**Age**				
Normal GMS	−0.025	0.184	-	−0.387; 0.336
Prediabetes	−0.663	0.284	α	−1.220; −0.107
T2DM	−0.755	0.181	α	−1.111; −0.399
**High SBP**				
Belgium	6.455	3.988	-	−1.366; 14.275
The Netherlands	−5.249	2.992	-	−11.117; 0.620
**History of CVD**				
Belgium	4.518	4.315	-	12.982; 0.373
The Netherlands	−10.756	3.431	α	−17.485; −4.027
**CAP difference at average age**				
CAP difference between prediabetes and normal GMS at the average age	−25.342	3.101	α	−31.424; −19.260
CAP difference between T2DM and normal GMS at the average age	−10.422	2.675	α	−15.668; −5.177
CAP difference between prediabetes and T2DM at the average age	14.920	3.115	α	8.810; 21.029

The continuous predictors were mean-centred; for age, the mean was 65.63 years, and for BMI, the mean was 29.2 kg/m^2^. Sex coded as 0 = male and 1 = female. Abbreviations: BMI: body mass index, CI: confidence interval, CVD: cardiovascular disease, SBP: systolic blood pressure, SE: standard error, T2DM: type 2 diabetes mellitus.

## Data Availability

Data are available from The Maastricht Study for any researcher who meets the criteria for access to confidential data; the corresponding author may be contacted to request data.

## Supplementary Materials

The following supporting information can be downloaded at: https://www.mdpi.com/article/10.3390/biomedicines13112821/s1, Table S1: Detailed overview of the prevalence of steatosis, fibrosis and GMS status based on country, centres and primary care practices; Table S2: Comparability of glucose metabolism status (GMS) across cohorts; Table S3: Detailed description of medication use and diabetes duration for people with T2DM in Belgium and the Netherlands; Table S4: Linear regression analysis for risk factors of steatosis in the prediabetes, T2DM, and control MASLD population with Belgium as a reference category for country; Table S5: Linear regression analysis for risk factors of steatosis in the prediabetes, T2DM, and control MASLD population with prediabetes as a reference category for GMS; Table S6: Linear regression analysis for risk factors of steatosis in the prediabetes, T2DM, and control MASLD population with T2DM as a reference category for GMS; Table S7: Linear regression analysis for risk factors of steatosis in the prediabetes, T2DM, and control MASLD population with the Netherlands as a reference category for country; Table S8: Linear regression analysis for risk factors of steatosis in the prediabetes, T2DM, and control MASLD population with Belgium as a reference category for the country; Table S9: Linear regression analysis for risk factors of steatosis in the prediabetes, T2DM, and control MASLD population, with prediabetes as a reference category for GMS: title; Table S10: Linear regression analysis for risk factors of steatosis in the prediabetes, T2DM, and control MASLD population with T2DM as a reference category for GMS; Table S11: Linear regression analysis for risk factors of steatosis in the prediabetes, T2DM, and control MASLD population with the Netherlands as a reference category for country; Table S12: Estimated marginal means (EMM) for CAP by CVD status per country, evaluated at mean Age and BMI values, when adjusting for statins use; Table S13: Estimated marginal means (EMM) for CAP by CVD status per country, evaluated at mean Age and BMI values, without adjusting for statins use; Figure S1: Age-related differences in CAP across GMS; Figure S2: Quadratic effect of BMI and raw BMI on CAP; Figure S3: Overview of the most important findings.

## Appendix A

Members of MASLD Research Group are listed as follows:

Y. Kockaerts ^1^, I. Lowyck ^1^, K. Stinkens ^1^, H. Van Leeuwen-Wintjens ^1^, L. Vernijns ^2^, J. Germaux ^3^, T. Meyers ^4^, J. Gyselaers ^5^, S. Pietermans ^6^, R. Bollen ^7^, A. Robaeys ^8^, C. Gilio ^9^, R. Remmen ^10^, K. de Munck ^11^, E. Rubens ^11^, K. Joris ^11^, M. Brouwers ^12,13,14^, S Meex ^15,16^ and A. Kroon ^15^

1 Ziekenhuis Oost-Limburg, Department of Endocrinology, Genk, Belgium
2 Huisartsenbox, Genk, Belgium
3 Huisartsenpraktijk Medi-Mine, Genk, Belgium
4 Groepspraktijk Luce, Genk, Belgium
5 Gezondheidscentrum Sirona vzw, Genk, Belgium
6 Groepspraktijk De Dam, Maasmechelen, Belgium
7 W-Care Hoeselt, Hoeselt, Belgium
8 Huisartsen Termolen, Zonhoven, Belgium
9 Huisarts Gilio, Maasmechelen, Belgium
10 University of Antwerp, Maatschappelijke Gezondheidszorg en Eerstelijnzorg, Wilrijk, Belgium
11 Antwerp University Hospital, Department of Gastroenterology and Hepatology, Antwerp, Belgium
12 MUMC+, Department of Endocrinology, Maastricht University Medical Centre, The Netherlands
13 Cardiovascular Research Institute Maastricht (CARIM), Maastricht University, Maastricht, The Netherlands
14 Care and Public Health Research Institute (CAPHRI), Maastricht University, Maastricht, The Netherlands
15 Department Clinical Chemistry, Division Diagnostic Laboratory, Maastricht University Medical Center, The Netherlands
