# Supplementary material for: Prevalence and Risk Factors of MASLD in Prediabetes and Type 2 Diabetes Mellitus in Belgium and The Netherlands
Source: Biomedicines. 2025 Nov 19;13(11):2821. doi: 10.3390/biomedicines13112821 (PMC12649957; doi:10.3390/biomedicines13112821)
Supplement: Supplementary file 1 [file biomedicines-13-02821-s001.zip › biomedicines-3957233-supplementary.pdf]

Supplemental materials

Table S1: Detailed overview of the prevalence of steatosis, fibrosis and GMS status based on country, centres and primary care practices.

	**Belgium**												**Netherlands**
	**Primary care**											**Secondary care (n=297)**	
	**Limburg (n=235)**									**Ghent (n=155)**	**Antwerp (n=124)**		
	**Total**	**1 (n=28)**	**2 (n=56)**	**3 (n=3)**	**4 (n=25)**	**5 (n=10)**	**6 (n=5)**	**7 (n=18)**	**8 (n=59)**				
**Steatosis**	212 (90.2)	23 (82.1)	51 (91.1)	3 (100.0)	22 (88.0)	37 (92.5)	5 (100.0)	17 (94.4)	53 (89.8)	139 (89.7)	112 (90.3	247 (83.2)	925 (82.8)
**Fibrosis >8kPa**	20 (8.5)	2 (7.1)	6 (10.7)	0 (0.0)	2 (8.0)	4 (10.0)	0 (0.0)	1 (5.6)	5 (8.5)	35 (22.6)	11 (8.9)	69 (23.2)	30 (2.7)
**Normal GMS**	139 (59.1)	18 (64.3)	32 (57.1)	3 (100.0)	16 (64.0)	23 (57.5)	5 (100.0)	9 (50.0)	33 (55.9)	78 (50.3)	62 (50.0)	0 (0.0)	459 (41.1)
**Prediab**	54 (23.0)	8 (28.6)	17 (30.4)	0 (0.0)	3 (12.0)	9 (22.5)	0 (0.0)	3 (16.7)	14 (23.7)	28 (18.1)	62 (16.1)	0 (0.0)	269 (24.1)
**T2DM**	42 (17.9)	2 (7.1)	7 (12.5)	0 (0.0)	6 (24.0)	8 (20.0)	0 (0.0)	6 (33.3)	12 (20.3)	49 (31.6)	42 (33.9)	297 (100)	389 (34.8)

Data are presented as numbers (%). Primary care practice: 1 = Zonhoven (Ter Molen), 2 = Huisartsenbox, 3 = Medi-Mine, 4 = Gezondheidscentrum Sirona, 5 = Groepspraktijk Luce, 6 = Dr. Gilio, 7 = Groepspraktijk De Dam, 8=W-Care.

Table S2: Comparability of glucose metabolism status (GMS) across cohorts.

Component	Belgium	Netherlands
**Prediabetes**		
Impaired fasting glucose	110-125 mg/dL	FPG between 110 and 125 mg/dL **and** 2h-PFG <140 mg/dL
Impaired glucose tolerance	Not used directly	FPG <126 mg/dL **and** 2h-PFG ≥140 mg/dl and <200 mg/dL
HbA1c	6-6.4%	Not used
**T2DM**		
Impaired fasting glucose	Fasting glucose ≥ 126 mg/dL or based on EPR	FPG ≥ 126 mg/dL
Impaired glucose tolerance	Not used directly	2h FPG ≥200 mg/dL
T2DM by meds	Use of glucose-lowering drugs	Use of glucose-lowering drugs
**MetS**		
Waist circumference	≥94 cm men / ≥80 cm women	≥94 cm men / ≥80 cm women
Blood pressure	≥130/85 mmHg or antihypertensive meds<br>Blood pressure was only measured once.	≥130/85 mmHg or antihypertensive meds<br>Measured thrice, and the average was used.
Triglycerides	≥150 mg/dL	≥150 mg/dL
High-density lipoprotein cholesterol	<40 mg/dL men / <50 mg/dL women	<40 mg/dL men / <50 mg/dL women
Fasting glucose	≥100 mg/dL or diabetes	≥100 mg/dL or diabetes

A harmonisation table was created mapping all relevant components, including fasting glucose, OGTT results, HbA1c, use of glucose-lowering medications, and individual metabolic syndrome (MetS) criteria (waist circumference, blood pressure, triglycerides, HDL cholesterol, fasting glucose)

Abbreviations: HbA1c: haemoglobin A1c, EPR: electronic patient record, FPG: fasting plasma glucose.

Table S3: Detailed description of medication use and diabetes duration for people with T2DM in Belgium and the Netherlands.

	T2DM Belgium (n=430)	T2DM Netherlands (n=389)	p
Glucose-lowering medication (all types)	362 (94.8)	277 (71.2)	<0.001
Metformin	280 (65.1)	248 (63.8)	0.009
Dipeptidyl peptidase 4 (DPP4)	19 (4.4)	37 (9.5)	0.032
Thiazolidines	0 (0.0)	1 (0.3)	-
Sulfonylurea derivates	87 (20.2)	92 (23.7)	0.579
Sodium-glucose cotransporter 2 (SGLT2)	49 (11.4)	40 (10.3)	0.321
Glucagon-like-peptide 1 (GLP-1) agonists	95 (22.1)	19 (4.9)	<0.001
Insulin	150 (34.9)	69 (17.7)	<0.001
Diabetes duration	7.0 [11] (n=337)	11 [18] (n=250)	-

Data are presented as numbers (%).

Table S4: Linear regression analysis for risk factors of steatosis in the prediabetes, T2DM, and control MASLD population with Belgium as a reference category for country.

Variable	B	Std. Error	Beta	t	Sig.	95% CI Lower	95% CI Upper	Tolerance	VIF
(Intercept)	289.072	4.317	–	66.962	<0.001	280.605	297.538	–	–
Age (centred)	-0.025	0.184	-0.004	-0.137	0.891	-0.387	0.336	0.371	2.695
Prediabetes	-25.342	3.101	-0.186	-8.172	<0.001	-31.424	-19.260	0.763	1.310
T2DM	-10.422	2.675	-0.095	-3.897	<0.001	-15.668	-5.177	0.660	1.514
Sex	-7.445	2.238	-0.068	-3.327	0.001	-11.834	-3.056	0.935	1.069
BMI (centred)	5.184	0.284	0.452	18.248	<0.001	4.627	5.741	0.642	1.559
BMI^2	-0.077	0.031	-0.057	-2.461	0.014	-0.138	-0.016	0.738	1.355
MetS	13.725	2.686	0.122	5.109	<0.001	8.456	18.994	0.688	1.453
High SBP	6.455	3.988	0.056	1.619	0.106	-1.366	14.275	0.325	3.073
Country	15.256	4.417	0.138	3.454	<0.001	6.593	23.919	0.246	4.063
History of CVD	4.518	4.315	0.034	1.047	0.295	-3.945	12.982	0.373	2.683
**Interaction terms**									
Age*prediabetes	-0.638	0.332	-0.046	-1.923	0.055	-1.289	0.013	0.677	1.478
Age*T2DM	-0.730	0.249	-0.084	-2.933	0.003	-1.218	-0.242	0.474	2.108
High SBP*country	-11.703	4.902	-0.104	-2.388	0.017	-21.317	-2.090	0.206	4.845
History of CVD*country	-15.274	5.415	-0.094	-2.820	0.005	-25.895	-4.653	0.353	2.835

The continuous predictors were mean-centred; for age, the mean was 65.63 years, and for BMI, the mean was 29.2 kg/m². Sex coded as 0=male and 1=female.
Abbreviations: BMI: body mass index, CI: confidence interval, CVD: cardiovascular disease, SBP: systolic blood pressure, SE: standard error, T2DM: type 2 diabetes mellitus.

Table S5: Linear regression analysis for risk factors of steatosis in the prediabetes, T2DM, and control MASLD population with prediabetes as a reference category for GMS.

Variable	B	Std. Error	Beta	t	Sig.	95% CI Lower	95% CI Upper
(Intercept)	263.73	4.899		53.835	<.001	254.122	273.338
Age (centred)	-0.663	0.284	-0.117	-2.339	0.019	-1.22	-0.107
Normal GMS	25.342	3.101	0.228	8.172	<.001	19.26	31.424
T2DM	14.92	3.115	0.136	4.79	<.001	8.81	21.029
Sex	-7.445	2.238	-0.068	-3.327	<.001	-11.834	-3.056
BMI (centred)	5.184	0.284	0.452	18.248	<.001	4.627	5.741
BMI^2	-0.077	0.031	-0.057	-2.461	0.014	-0.138	-0.016
MetS	13.725	2.686	0.122	5.109	<.001	8.456	18.994
High SBP	6.455	3.988	0.056	1.619	0.106	-1.366	14.275
Country	15.256	4.417	0.138	3.454	<.001	6.593	23.919
History of CVD	4.518	4.315	0.034	1.047	0.295	-3.945	12.982
**Interaction terms**							
Age*prediabetes	0.638	0.332	0.071	1.923	0.055	-0.013	1.289
Age*T2DM	-0.092	0.329	-0.011	-0.279	0.78	-0.737	0.553
High SBP*country	-11.703	4.902	-0.104	-2.388	0.017	-21.317	-2.09
History of CVD*country	-15.274	5.415	-0.094	-2.82	0.005	-25.895	-4.653

The continuous predictors were mean-centred; for age, the mean was 65.63 years, and for BMI, the mean was 29.2 kg/m². Sex coded as 0=male and 1=female.
Abbreviations: BMI: body mass index, CI: confidence interval, CVD: cardiovascular disease, SBP: systolic blood pressure, SE: standard error, T2DM: type 2 diabetes mellitus.

Table S6: Linear regression analysis for risk factors of steatosis in the prediabetes, T2DM, and control MASLD population with T2DM as a reference category for GMS.

Variable	B	Std. Error	Beta	t	Sig.	95% CI Lower	95% CI Upper
(Intercept)	278.649	4.472		62.314	<.001	269.879	287.42
Age (centred)	-0.755	0.181	-0.133	-4.162	<.001	-1.111	-0.399
Normal GMS	10.422	2.675	0.094	3.897	<.001	5.177	15.668
Prediabetes	-14.92	3.115	-0.109	-4.79	<.001	-21.029	-8.81
Sex	-7.445	2.238	-0.068	-3.327	<.001	-11.834	-3.056
BMI (centred)	5.184	0.284	0.452	18.248	<.001	4.627	5.741
BMI^2	-0.077	0.031	-0.057	-2.461	0.014	-0.138	-0.016
MetS	13.725	2.686	0.122	5.109	<.001	8.456	18.994
High SBP	6.455	3.988	0.056	1.619	0.106	-1.366	14.275
Country	15.256	4.417	0.138	3.454	<.001	6.593	23.919
History of CVD	4.518	4.315	0.034	1.047	0.295	-3.945	12.982
**Interaction terms**							
Age*prediabetes	0.73	0.249	-0.081	2.933	0.003	0.242	1.218
Age*normal GMS	0.092	0.329	0.007	0.279	0.78	-0.553	0.737
High SBP*country	-11.703	4.902	-0.104	-2.388	0.017	-21.317	-2.09
History of CVD*country	-15.274	5.415	-0.094	-2.82	0.005	-25.895	-4.653

The continuous predictors were mean-centred; for age, the mean was 65.63 years, and for BMI, the mean was 29.2 kg/m². Sex coded as 0=male and 1=female.
Abbreviations: BMI: body mass index, CI: confidence interval, CVD: cardiovascular disease, SBP: systolic blood pressure, SE: standard error, T2DM: type 2 diabetes mellitus.

Table S7: Linear regression analysis for risk factors of steatosis in the prediabetes, T2DM, and control MASLD population with the Netherlands as a reference category for country.

Variable	B	Std. Error	Beta	t	Sig.	95% CI Lower	95% CI Upper
(Intercept)	304.328	3.117	–	97.643	.000	298.215	310.440
Age (centred)	-0.025	0.184	-0.004	-0.137	0.891	-0.387	0.336
Prediabetes	-25.342	3.101	-0.186	-8.172	<.001	-31.424	-19.260
T2DM	-10.422	2.675	-0.095	-3.897	<.001	-15.668	-5.177
Sex	-7.445	2.238	-0.068	-3.327	<.001	-11.834	-3.056
BMI (centred)	5.184	0.284	0.452	18.248	<.001	4.627	5.741
BMI^2	-0.077	0.031	-0.057	-2.461	0.014	-0.138	-0.016
MetS	13.725	2.686	0.122	5.109	<.001	8.456	18.994
High SBP	-5.249	2.992	-0.046	-1.754	0.080	-11.117	0.620
Country	-15.256	4.417	-0.138	-3.454	<.001	-23.919	-6.593
History of CVD	-10.756	3.431	-0.081	-3.135	0.002	-17.485	-4.027
**Interaction terms**							
Age*prediabetes	-0.638	0.332	-0.046	-1.923	0.055	-1.289	0.013
Age*T2DM	-0.730	0.249	-0.084	-2.933	0.003	-1.218	-0.242
High SBP*country	11.703	4.902	0.099	2.388	0.017	2.090	21.317
History of CVD*country	15.274	5.415	0.078	2.820	0.005	4.653	25.895

The continuous predictors were mean-centred; for age, the mean was 65.63 years, and for BMI, the mean was 29.2 kg/m². Sex coded as 0=male and 1=female.
Abbreviations: BMI: body mass index, CI: confidence interval, CVD: cardiovascular disease, SBP: systolic blood pressure, SE: standard error, T2DM: type 2 diabetes mellitus.

Table S8: Linear regression analysis for risk factors of steatosis in the prediabetes, T2DM, and control MASLD population with Belgium as a reference category for the country.

Variable	B	Std. Error	Beta	t	Sig.	95% CI Lower	95% CI Upper	Tolerance	VIF
(Intercept)	290.726	4.386		66.292	.000	282.125	299.327		
Age (centred)	-.003	.185	-.001	-.018	.985	-.366	.360	.368	2.718
Prediabetes	-25.158	3.094	-.184	-8.133	<.001	-31.225	-19.091	.762	1.311
T2DM	-9.587	2.747	-.087	-3.489	<.001	-14.975	-4.198	.624	1.603
Sex	-7.849	2.242	-.072	-3.500	<.001	-12.246	-3.451	.928	1.078
BMI (centred)	5.310	.285	.462	18.622	<.001	4.751	5.869	.636	1.573
BMI^2	-.088	.031	-.065	-2.803	.005	-.149	-.026	.733	1.365
MetS	13.761	2.709	.122	5.080	<.001	8.448	19.074	.674	1.483
High SBP	6.091	3.992	.053	1.526	.127	-1.738	13.921	.324	3.091
Country	14.981	4.428	.135	3.383	<.001	6.297	23.665	.244	4.094
History of CVD	5.153	4.318	.039	1.194	.233	-3.315	13.622	.371	2.696
**Lipid-lowering medication, statins: HMG CoA reductase inhibitors**	-3.498	2.430	-.032	-1.440	.150	-8.264	1.267	.786	1.272
**Interaction terms**									
Age*prediabetes	-.633	.331	-.046	-1.912	.056	-1.282	.016	.676	1.479
Age*T2DM	-.781	.249	-.090	-3.136	.002	-1.270	-.293	.474	2.108
High SBP*country	-11.387	4.905	-.101	-2.321	.020	-21.007	-1.767	.205	4.876
History of CVD*country	-14.990	5.428	-.093	-2.762	.006	-25.635	-4.345	.349	2.865

The continuous predictors were mean-centred; for age, the mean was 65.63 years, and for BMI, the mean was 29.2 kg/m². Sex coded as 0=male and 1=female.
Abbreviations: BMI: body mass index, CI: confidence interval, CVD: cardiovascular disease, SBP: systolic blood pressure, SE: standard error, T2DM: type 2 diabetes mellitus.

Table S9: Linear regression analysis for risk factors of steatosis in the prediabetes, T2DM, and control MASLD population, with prediabetes as a reference category for GMS.

Variable	B	Std. Error	Beta	t	Sig.	95% CI Lower	95% CI Upper
(Intercept)	265.568	4.971		53.424	.000	255.819	275.317
Age (centred)	-.636	.283	-.112	-2.246	.025	-1.191	-.081
Normal GMS	25.158	3.094	.226	8.133	<.001	19.091	31.225
T2DM	15.572	3.156	.142	4.934	<.001	9.382	21.761
Sex	-7.849	2.242	-.072	-3.500	<.001	-12.246	-3.451
BMI (centred)	5.310	.285	.462	18.622	<.001	4.751	5.869
BMI^2	-.088	.031	-.065	-2.803	.005	-.149	-.026
MetS	13.761	2.709	.122	5.080	<.001	8.448	19.074
High SBP	6.091	3.992	.053	1.526	.127	-1.738	13.921
Country	14.981	4.428	.135	3.383	<.001	6.297	23.665
History of CVD	5.153	4.318	.039	1.194	.233	-3.315	13.622
Lipid-lowering medication, statins: HMG CoA reductase inhibitors	-3.498	2.430	-.032	-1.440	.150	-8.264	1.267
Interaction terms							
Age*prediabetes	.633	.331	.070	1.912	.056	-.016	1.282
Age*T2DM	-.149	.329	-.017	-.453	.651	-.793	.495
High SBP*country	-11.387	4.905	-.101	-2.321	.020	-21.007	-1.767
History of CVD*country	-14.990	5.428	-.093	-2.762	.006	-25.635	-4.345

The continuous predictors were mean-centred; for age, the mean was 65.63 years, and for BMI, the mean was 29.2 kg/m². Sex coded as 0=male and 1=female.
Abbreviations: BMI: body mass index, CI: confidence interval, CVD: cardiovascular disease, SBP: systolic blood pressure, SE: standard error, T2DM: type 2 diabetes mellitus.

Table S10: Linear regression analysis for risk factors of steatosis in the prediabetes, T2DM, and control MASLD population with T2DM as a reference category for GMS.

Variable	B	Std. Error	Beta	t	Sig.	95% CI Lower	95% CI Upper
(Intercept)	281.140	4.685		60.007	.000	271.951	290.328
Age (centred)	-.785	.182	-.138	-4.309	<.001	-1.142	-.428
Normal GMS	9.587	2.747	.086	3.489	<.001	4.198	14.975
Prediabetes	-15.572	3.156	-.114	-4.934	<.001	-21.761	-9.382
Sex	-7.849	2.242	-.072	-3.500	<.001	-12.246	-3.451
BMI (centred)	5.310	.285	.462	18.622	<.001	4.751	5.869
BMI^2	-.088	.031	-.065	-2.803	.005	-.149	-.026
MetS	13.761	2.709	.122	5.080	<.001	8.448	19.074
High SBP	6.091	3.992	.053	1.526	.127	-1.738	13.921
Country	14.981	4.428	.135	3.383	<.001	6.297	23.665
History of CVD	5.153	4.318	.039	1.194	.233	-3.315	13.622
**Lipid-lowering medication, statins: HMG CoA reductase inhibitors**	-3.498	2.430	-.032	-1.440	.150	-8.264	1.267
**Interaction terms**							
Age*prediabetes	.781	.249	.087	3.136	.002	.293	1.270
Age*T2DM	.149	.329	.011	.453	.651	-.495	.793
High SBP*country	-11.387	4.905	-.101	-2.321	.020	-21.007	-1.767
History of CVD*country	-14.990	5.428	-.093	-2.762	.006	-25.635	-4.345

The continuous predictors were mean-centred; for age, the mean was 65.63 years, and for BMI, the mean was 29.2 kg/m². Sex coded as 0=male and 1=female.
Abbreviations: BMI: body mass index, CI: confidence interval, CVD: cardiovascular disease, SBP: systolic blood pressure, SE: standard error, T2DM: type 2 diabetes mellitus.

Table S11: Linear regression analysis for risk factors of steatosis in the prediabetes, T2DM, and control MASLD population with the Netherlands as a reference category for country.

Variable	B	Std. Error	Beta	t	Sig.	95% CI Lower	95% CI Upper
(Intercept)	305.707	3.149		97.083	.000	299.531	311.883
Age (centred)	-.003	.185	-.001	-.018	.985	-.366	.360
Prediabetes	-25.158	3.094	-.184	-8.133	<.001	-31.225	-19.091
T2DM	-9.587	2.747	-.087	-3.489	<.001	-14.975	-4.198
Sex	-7.849	2.242	-.072	-3.500	<.001	-12.246	-3.451
BMI (centred)	5.310	.285	.462	18.622	<.001	4.751	5.869
BMI^2	-.088	.031	-.065	-2.803	.005	-.149	-.026
MetS	13.761	2.709	.122	5.080	<.001	8.448	19.074
High SBP	-5.296	2.986	-.046	-1.774	.076	-11.151	.560
Country	-14.981	4.428	-.135	-3.383	<.001	-23.665	-6.297
History of CVD	-9.837	3.476	-.074	-2.830	.005	-16.655	-3.019
Lipid-lowering medication, statins: HMG CoA reductase inhibitors	-3.498	2.430	-.032	-1.440	.150	-8.264	1.267
Interaction terms							
Age*prediabetes	-.633	.331	-.046	-1.912	.056	-1.282	.016
Age*T2DM	-.781	.249	-.090	-3.136	.002	-1.270	-.293
High SBP*country	11.387	4.905	.096	2.321	.020	1.767	21.007
History of CVD*country	14.990	5.428	.076	2.762	.006	4.345	25.635

The continuous predictors were mean-centred; for age, the mean was 65.63 years, and for BMI, the mean was 29.2 kg/m². Sex coded as 0=male and 1=female.
Abbreviations: BMI: body mass index, CI: confidence interval, CVD: cardiovascular disease, SBP: systolic blood pressure, SE: standard error, T2DM: type 2 diabetes mellitus. **T**

Table S12: Estimated marginal means (EMM) for CAP by CVD status per country, evaluated at mean Age and BMI values, when adjusting for statins use

Country	CVD status	EMM of CAP (dB/m)	SE	95% CI
**Belgium**	No History of CVD	283	2.48	279; 288
	History of CVD	289	4.13	280; 297
**Netherlands**	No History of CVD	293	1.91	289; 296
	History of CVD	283	3.22	277; 289

Table S13: Estimated marginal means (EMM) for CAP by CVD status per country, evaluated at mean Age and BMI values, without adjusting for statins use

Country	CVD status	EMM of CAP (dB/m)	SE	95% CI
**Belgium**	No History of CVD	284	2.47	279; 288
	History of CVD	288	4.13	280; 296
**Netherlands**	No History of CVD	293	1.85	289; 297
	History of CVD	282	3.22	276; 288

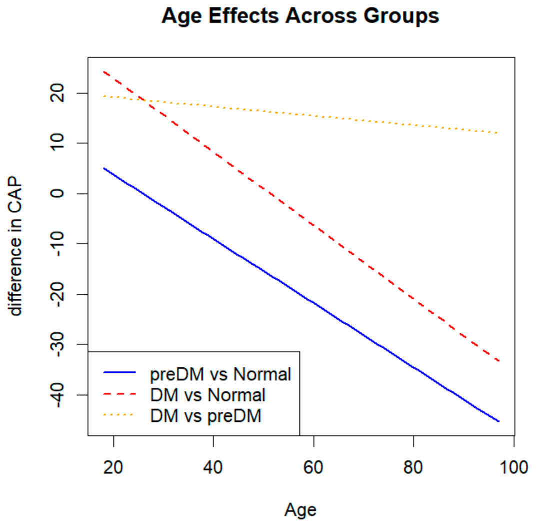

Figure S1: Age-related differences in CAP across GMS.

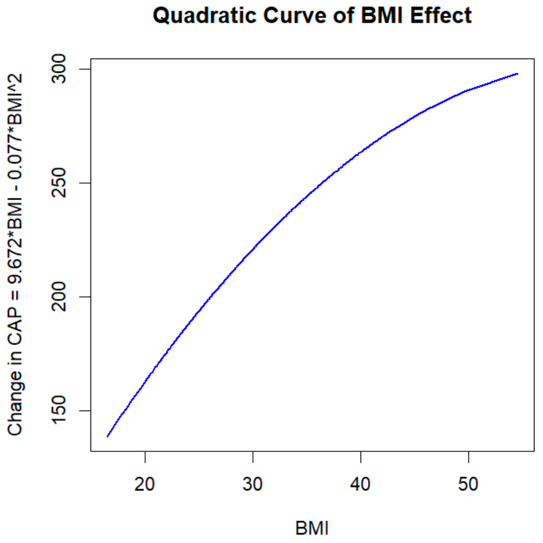

Figure S2: Quadratic effect of BMI and raw BMI on CAP.

	Belgium		The Netherlands		p[1]	p[2]
	T2DM (n=430)	Prediabetes (n=102)	T2DM (n=389)	Prediabetes (n=269)		
VCTE™ (kPa)	6.6 [5.1]	4.9 [2.1]	5.3 [2.3]	4.4 [1.7]	α	-
Significant fibrosis	166 (38.6)	12 (11.8)	38 (9.8)	4 (1.5)	α	α
Advanced fibrosis	78 (18.1)	0 (0.0)	14 (3.6)	1 (0.4)	α	-
CAP™ (dB/m)	312 [81]	270.5±50.1	294 [93]	265.5±58.3	α	-
MASLD	354 (82.3)	74 (72.5)	290 (74.6)	173 (64.3)	α	-

Variable	β	SE	p	95%CI
Variables not part of any interactions				
Sex	-7.445	2.238	α	-11.834; -3.056
BMI	5.184	0.284	α	4.627; 5.741
MetS	13.725	2.686	α	8.456; 18.994
Effect of interaction terms on CAP				
History of CVD				
Belgium	4.518	4.315	-	12.982; 0.373
the Netherlands	-10.756	3.431	α	-17.485; -4.027

Figure S3: Overview of the most important findings.
